# Transspinal stimulation increases motoneuron output of multiple segments in human spinal cord injury

**DOI:** 10.1371/journal.pone.0213696

**Published:** 2019-03-07

**Authors:** Lynda M. Murray, Maria Knikou

**Affiliations:** 1 Klab4Recovery Research Laboratory, Department of Physical Therapy, College of Staten Island, The City University of New York, Staten Island, New York, United States of America; 2 PhD Program in Biology and Collaborative Neuroscience Program, Graduate Center of The City University of New York, New York, New York, United States of America; Szegedi Tudomanyegyetem, HUNGARY

## Abstract

Targeted neuromodulation strategies that strengthen neuronal activity are in great need for restoring sensorimotor function after chronic spinal cord injury (SCI). In this study, we established changes in the motoneuron output of individuals with and without SCI after repeated noninvasive transspinal stimulation at rest over the thoracolumbar enlargement, the spinal location of leg motor circuits. Cases of motor incomplete and complete SCI were included to delineate potential differences when corticospinal motor drive is minimal. All 10 SCI and 10 healthy control subjects received daily monophasic transspinal stimuli of 1-ms duration at 0.2 Hz at right soleus transspinal evoked potential (TEP) subthreshold and suprathreshold intensities at rest. Before and two days after cessation of transspinal stimulation, we determined changes in TEP recruitment input-output curves, TEP amplitude at stimulation frequencies of 0.1, 0.125, 0.2, 0.33 and 1.0 Hz, and TEP postactivation depression upon transspinal paired stimuli at interstimulus intervals of 60, 100, 300, and 500 ms. TEPs were recorded at rest from bilateral ankle and knee flexor/extensor muscles. Repeated transspinal stimulation increased the motoneuron output over multiple segments. In control and complete SCI subjects, motoneuron output increased for knee muscles, while in motor incomplete SCI subjects motoneuron output increased for both ankle and knee muscles. In control subjects, TEPs homosynaptic and postactivation depression were present at baseline, and were potentiated for the distal ankle or knee flexor muscles. TEPs homosynaptic and postactivation depression at baseline depended on the completeness of the SCI, with minimal changes observed after transspinal stimulation. These results indicate that repeated transspinal stimulation increases spinal motoneuron responsiveness of ankle and knee muscles in the injured human spinal cord, and thus can promote motor recovery. This noninvasive neuromodulation method is a promising modality for promoting functional neuroplasticity after SCI.

## Introduction

Targeted neuromodulation strategies that strengthen neuronal activity are in great need for restoring sensorimotor function after chronic spinal cord injury (SCI). Within this concept, several therapeutic approaches promoting neuromodulation and thereby neuroplasticity have been adapted over the last few decades [1–6]. One of these methods includes spinal cord stimulation delivered epidurally or transcutaneously to the lumbar spinal region, the location of the leg motor circuits. Evidence supports for some recovery of standing and walking ability during or after epidural delivery of electrical current to the spinal cord of individuals with complete SCI that received concomitantly a variable number of locomotor training sessions [7–14]. Epidural stimulation also promoted bladder control, cardiovascular function, respiration and cough in human SCI [14–18]. Similarly, transcutaneous spinal cord (termed here transspinal) stimulation at low and high frequencies strengthens corticomotoneuronal connectivity, decreases hyperreflexia, improves bladder function, seated postural control, and autonomic cardiovascular function [19–23]. Step-like movements produced by transspinal stimulation [24], further support its ability to modulate spinal locomotor circuits in persons with paralysis. However, the effect of repeated low-frequency transspinal stimulation on the responsiveness of spinal motoneurons in individuals with SCI remains largely undetermined.

At the neuronal level, transspinal stimulation induces synaptic activity and intrinsic polarization of motoneurons [25], and decreases the magnitude of dendritic persistent inward currents that are related to hyperreflexia and spasms after SCI [26–28]. Further, transspinal stimulation activates receptors required for long-term potentiation-like actions of neurotrophins that mediate plasticity [29,30]. At the circuitry level, single-pulse transspinal stimulation generates transspinal evoked potentials (TEPs) simultaneously in bilateral lower limb muscles in individuals with and without SCI with distinct neurophysiological characteristics regarding their latency, duration, shape, and spinal integration [19,24,31–34]. TEPs exhibit a sigmoid function at increasing stimulation intensities, which coincides with bilateral leg extension, supporting the depolarization of motoneurons across multiple spinal segments that has been linked to stimulation-induced formation and retention of motor memories [35].

Collectively, the objectives of this study were to establish the effects of daily low-frequency transspinal stimulation on spinal motoneuron output and spinal inhibition of multiple spinal segments. We hypothesized that low-frequency transspinal stimulation at levels that produces motoneuron depolarization over multiple segments strengthens spinal connections and thus increases motoneuron output. We further hypothesized that motoneuron responsiveness will be greater in individuals with incomplete SCI compared to that observed in individuals with complete SCI. To test our hypotheses, the TEP recruitment input-output curves, TEP depression at low stimulation frequencies, termed here homosynaptic depression, and TEP depression in response to transspinal paired stimuli, termed here postactivation depression, recorded bilateral from ankle and knee flexor/extensor muscles were assessed at rest before and two days after an average of 13 sessions of transspinal stimulation delivered at 0.2 Hz in people with and without SCI.

## Materials and methods

### Participants

All experimental and stimulation procedures were conducted in compliance with the Declaration of Helsinki after full Institutional Review Board (IRB) approval by the City University of New York IRB committee (IRB Number: 515055). A written informed consent was obtained from all participants before study enrollment. Participants were instructed to refrain from alcohol and caffeine consumption at least 24 hours before testing, and be cannabis-free for more than one week.

Ten individuals with chronic SCI (Table 1) and 10 individuals without SCI (5 female; 30.9 ± 14 years, mean ± SD) were enrolled in the study. Individuals with SCI were recruited based on the following criteria: age 18–65 years; chronic injury (more than 6 months after injury); and able to tolerate lying supine or sitting upright for at least 1 hour. Individuals with SCI were excluded if they presented with concomitant traumatic brain injury or stroke, cognitive impairment, not medically stable, or had contraindications to spinal stimulation (presence of an implanted object such as a pacemaker or pump). Five individuals with chronic SCI had a neurological deficit grade D on the American Spinal Injury Association Impairment Scale (AIS), 1 had AIS C, 2 had AIS B, and 2 had AIS A. The level of SCI ranged from C4 to T11. Individuals with complete SCI were included in order to assess potential changes when corticomotoneuronal control is minimal. Individuals without SCI had no history of neurological or musculoskeletal disorders.

**Table 1:** Characteristics and demographics of individuals with spinal cord injury (SCI).

ID	Gender	Age (yrs)	Post injury (yrs)	Level of injury			Motor score		Number of sessions	List of medication
					AIS	Cause of injury	LL	RL		
R01	M	51	3.5	C7	B	Ocean Wave-related	0	0	17	Baclofen 20 mg 4xD; Sertraline 60 mg 1xD; Oxybutynin 5 mg 3xD
R03	F	24	2	C6	D	Fall from height	20	5	11	Nitrofurantoin 100 mg 1xD; Amitriptyline 50 mg 1xD; Dextroamphetamine-Amphetamine 20 mg 2xD
R04	M	51	2	T5	D	Calcification of ligaments	18	18	15	Metaxalone 800 mg 4xD; Oxycodone 10–325 mg 4xD; Baclofen 200 mg 1-2xD; Diltiazem ER 240 mg 1xD; Valacyclovir 500 mg 3xW; Oxymorphone 40 mg 2xD; Omeprazole 20 mg 1xD
R06	M	36	4	T2	A	MVC	0	0	17	None
R07	F	39	16	T12	C	MVC	17	5	23	Baclofen 2 mg 2xD
R08	M	27	9	C7	B	MVC	0	0	18	Oxybutynin 10–15 mg 1xD
R09	F	19	5	T1	D	SX	21	12	15	None
R10	M	47	28	T7	A	GSW	0	0	14	None
R11	M	38	6	T9	D	GSW	23	23	18	Gabapentin 800 mg 3xD; Zenflox 200 mg 2xD; Baclofen 10 mg 3xD; Oxycodone-Acetaminophen 10 mg 3xD
R12	M	31	12	C6	D	MVC	25	17	18	None

Level of SCI corresponds to neurological level of injury. The American Spinal Injury Association Impairment Scale (AIS) is indicated for each subject based on sensory and motor evaluation per AIS guidelines. Motor scores (out of 25 maximal points for each leg) are indicated based on the manual muscle test of key muscles and evaluated as 0 = no contraction, 1 = flicker or trace of contraction, 2 = active movement with gravity eliminated, 3 = active movement against gravity, 4 = active movement against gravity and resistance, 5 = normal muscle power. The number of transspinal stimulation sessions given during the intervention is indicated for each participant. Medication was taken at similar times of day. MVC = Motor vehicle crash; SX = Surgery; GSW = Gunshot wound; xD = Times daily; xW = Times weekly; LL = left leg; RL = right leg.

### Noninvasive transspinal stimulation

The T10 spinous process was identified via palpation and in consolidation with anatomical landmarks. A single cathode electrode (Uni-Patch^TM^ EP84169, 10.2 × 5.1 cm^2^, MA, USA) was placed along the vertebrae equally between the left and right paravertebral sides. The electrode covered from T10 to L1-L2 vertebral levels. These vertebral levels correspond to L1 and S2 spinal segments and thus to the segmental innervation of the muscles from which action potentials were recorded in this study. A pair of interconnected reusable self-adhered anode electrodes (same type as the cathode), were placed on either side of the umbilicus or bilaterally on the iliac crests depending on the participant’s level of comfort or if the stimulation caused bladder discomfort [31,34]. The cathode and anode electrodes were connected to a constant current stimulator (DS7A, Digitimer, UK) that was triggered by Spike 2 scripts (Cambridge Electronics Design Ltd., UK). Optimal electrode placement was based on presence of TEPs in both proximal and distal lower limb muscles at low stimulation intensities, and presence of TEP depression upon paired transspinal stimuli. Once the optimal location was identified, the electrodes were affixed to the skin via Tegaderm transparent film (3M Healthcare, St Paul, Minnesota, USA). After each session, the stimulated skin area was marked by non-toxic skin pen and covered by Tegaderm film to ensure consistency of stimulation site across sessions.

During the intervention, transspinal stimulation was delivered on weekdays excluding holidays at 0.2 Hz with a single monophasic square-wave pulse of 1 ms duration while the participant lay supine at rest [36]. Knee and hip joints were flexed at 30° and ankles were supported in a neutral position. Individuals with SCI received an average of 16.6 ± 1 sessions for an average of 60 ± 2 min per session. Individuals without SCI received a total of 10.2 ± 0.2 sessions for an average of 40 ± 0.1 min per session. Stimulus intensity was established based on the intensity needed to determine the right soleus (SOL) TEP resting threshold at baseline. At baseline, the SOL TEP resting threshold intensity was 96.9 ± 24 and 28.9 ± 5.7 mA for individuals with and without SCI, respectively. Because the SOL TEP amplitude decreased after 10 to 15 min of continuous stimulation, we alternated the suprathreshold stimulation (15-min for SCI group; 10-min for control group) that evoked bilateral leg extension with subthreshold stimulation (5-min for both groups), similar to a stimulation paradigm we have previously utilized [20]. During each session, intensities ranged from 0.4 ± 0.1 to 4.3 ± 0.9 (2.2 ± 0.4) and 0.7 ± 0.1 to 10.3 ± 2.9 (6.4 ± 1.7) times the SOL TEP resting threshold for individuals with and without SCI, respectively. Transspinal stimulation was well tolerated by all participants, blood pressure remained stable, and no adverse events were encountered during the experiments or intervention.

### EMG recordings

Surface electromyographic (EMG) activity was recorded bilaterally by single bipolar differential electrodes (Motion Lab Systems Inc., Baton Rouge, Louisiana, USA) from the SOL, medial gastrocnemius (MG), peroneus longus (PL), tibialis anterior (TA), medial hamstring (MH), lateral hamstring (LH), rectus femoris (RF), and gracilis (GRC) muscles. EMG signals were amplified, filtered (10–1000 Hz), sampled at 2000 Hz via a 1401 plus (Cambridge Electronics Design Ltd., Cambridge, UK), and stored for offline analysis.

### Neurophysiological assessments before and after repeated transspinal stimulation

Measurements were performed at rest while lying supine, with transspinal stimulation electrodes placed using the same procedures for the intervention described above. Neurophysiological tests were performed before and two days after cessation of stimulation in individuals with and without SCI. Changes in multisegmental spinal motoneuron output were established based on the TEP recruitment input-output curve that was assembled at increasing intensities from below resting motor threshold until a plateau in the response was reached. Furthermore, TEPs from all muscles evoked at 1.3 ± 0.07 and 1.5 ± 0.16 times the SOL TEP threshold were recorded at 0.1, 0.125, 0.2, 0.33, and 1.0 Hz in order to establish changes in TEP homosynaptic depression in individuals with and without SCI, respectively. TEPs were also recorded upon paired transspinal stimuli randomly at the interstimulus intervals (ISIs) of 60, 100, 300 and 500 ms at 0.2 Hz in order to establish changes in TEPs postactivation depression for both subject groups. In all cases, 15 TEPs were recorded.

### Data analysis and statistics

Neurophysiological assessments were grouped separately for AIS C-D, AIS A-B, and healthy control subjects based on time of testing. TEPs recorded from ankle and knee muscles were measured as the area under the full-wave rectified waveform for identical time windows (Spike 2, CED Ltd., U.K.). The onset latency of TEPs recorded at 0.1 Hz upon single pulse at baseline were estimated for each participant based on the cumulative sum technique [37] on the unrectified waveform average and was compared between SCI and control subjects using a Student’s *t*-test.

TEPs recorded upon single-pulse transspinal stimulation at increasing stimulation intensities were normalized to the associated maximal TEP (TEPmax). The normalized TEPs were plotted against the non-normalized stimulation intensities and a Boltzmann sigmoid function (Eq 1; SigmaPlot 11, Systat Software Inc.) was fitted to the data [38,39]. In Eq 1, *m* is the slope parameter of the function, *S50-TEPmax* is the stimulus required to elicit a TEP equivalent to 50% of the TEPmax, and *s* is the TEP amplitude at a given stimulus value TEP. The TEP slope and stimuli corresponding to TEP threshold, 50% of the TEPmax, and maximal TEP were estimated based on Eqs 2, 3 and 4, respectively. The estimated parameters from the sigmoid fit were compared before and after transspinal stimulation using a Student’s *t*-test.

$$TEP(s)=\frac{TEPmax}{\left(1+\exp\left(m(s50-TEPmax-s)\right)\right)}$$ Eq 1

$$TEPslope=\frac{m\times TEPmax}{4}$$ Eq 2

$$TEPth\;stim=\frac{s-2}{m}$$ Eq 3

$$TEPmax\;stim=\frac{s+2}{m}$$ Eq 4

The predicted stimulation intensity from the sigmoid fit corresponding to S50-TEPmax obtained before transspinal stimulation was used to normalize the stimulation intensities for TEPs recorded before and after the intervention. Averages of normalized TEPs were calculated in steps of 0.05 from 0.3 up to 1.3 times the S50-TEPmax. The above described offline analysis was performed separately for each TEP input-output curve of each muscle for each participant assembled before and after transspinal stimulation. Repeated measures analysis of variance (rmANOVA) was then performed to the normalized TEPs grouped at multiples of S50-TEPmax before and after transspinal stimulation to establish the main effects of time grouped based on the normalized stimulation intensities. When a main effect was found, the Holm-Sidak *t*-tests for multiple comparisons were used to test for significant interactions between these factors.

To establish changes in homosynaptic depression, TEPs evoked at 0.125, 0.2, 0.33, and 1.0 Hz were expressed as a percentage of the mean amplitude of the homonymous TEP evoked at 0.1 Hz for each participant and muscle separately. To establish changes in postactivation depression, the TEP evoked by the second transspinal stimulus in the paired paradigm was normalized to the mean amplitude of the homonymous first TEP. This was done for TEPs recorded at ISIs of 60, 100, 300 and 500 ms for each subject and muscle separately. For both neurophysiological measures, rmANOVA was performed to establish the main effects of time. When a significant effect was found, Holm-Sidak *t*-tests for multiple comparisons were performed to establish interactions between time and ISI or between time and stimulation frequency. All values are presented as mean ± standard error of the mean unless otherwise stated. Values were considered significant with a *p* < .05.

## Results

### Characteristics of TEPs

**Fig 1:**
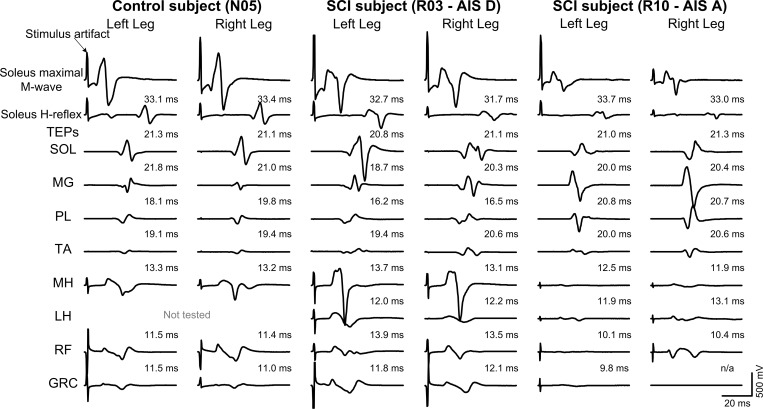
Transspinal evoked potentials (TEPs) in individuals with and without spinal cord injury (SCI). Non-rectified TEP waveform averages recorded (N = 15, elicited at 0.2 Hz) bilaterally from ankle and knee muscles (solid black lines) in a healthy control participant (N-005) and two individuals with SCI presenting with American Spinal Injury Association Impairment Scale (AIS) D (R-003) and AIS A (R-010), respectively. Latencies of the soleus (SOL) maximal M-wave and H-reflex (dashed lines) along with all TEPs across individuals are also presented. MG: medial gastrocnemius; PL: peroneus longus; TA: tibialis anterior; MH: medial hamstring; LH: lateral hamstring; RF: rectus femoris; GRC: gracilis.

Bilateral TEPs recorded from ankle and knee muscles from representative participants with and without SCI are indicated in Fig 1. TEPs are indicated along with the SOL maximal M-wave and SOL H-reflex for comparison of onset and shape between responses. Note that despite differences in the shape of TEPs across muscles, TEPs recorded from knee muscles have shorter latencies compared to the more distal ankle muscles, a phenomenon that was evident in both individuals with and without SCI. For individuals without SCI, TEPs were both biphasic and triphasic in nature for ankle muscles, whilst mostly triphasic for knee muscles. After incomplete SCI, TEPs were mostly triphasic with some polyphasic waveforms (see left RF in subject R03 and right RF in subject R10), and more turns were present within the waveforms. The latency of the SOL H-reflex and all TEPs recorded bilateral from ankle and knee muscles from all participants (N = 20) upon single pulses at 0.1 Hz are presented in Table 2. Latencies observed in individuals without SCI are consistent to those we have previously reported [31,34,40]. TEPs were present at similar latencies in the left and right leg muscles between subject groups. Note that the SOL TEP latency is approximately two thirds the SOL H-reflex latency for participants with (20.1 ± 0.5 ms; 32 ± 0.4 ms; 63%; based on grouped data) or without SCI (20.1 ± 0.4 ms; 31.5 ± 0.5 ms; 64%). These results suggest that SOL H-reflexes and SOL TEPs appear at similar latencies in individuals with and without SCI.

**Table 2:** Transspinal evoked potential (TEP) onset latency (ms) in individuals with and without spinal cord injury (SCI).

	Control Subjects		AIS C-D		AIS A-B	
	Left	Right	Left	Right	Left	Right
SOL H-reflex	31.6±0.81	31.4±0.62	31.5±0.40	31.0±0.39	33.3±1.12	33.4±0.27
SOL TEP	20.2±0.49	20.0±0.55	19.4±0.82	18.8±0.62	22.0±0.45	21.2±0.60
MG TEP	18.5±0.88	18.7±0.67	18.7±0.72	19.4±0.47	18.6±1.26	18.7±1.54
PL TEP	17.0±0.61	17.0±0.68	16.3±0.72	16.1±0.84	18.9±1.44	18.8±1.71
TA TEP	18.7±0.65	18.9±0.57	18.1±0.48	18.0±0.68	20.0±0.59	20.7±0.96
MH TEP	13.1±0.31	13.1±0.32	12.5±0.59	12.2±0.55	12.6±0.77	12.3±0.66
LH TEP	12.9±0.38	13.2±0.39	12.6±0.45	12.4±0.26	12.9±0.68	13.7±0.39
RF TEP	11.4±0.77	*11*.*9±0*.*56*	10.4±1.19	*9*.*5±1*.*10*	9.4±0.47	*9*.*8±0*.*24*
GRC TEP	11.6±0.49	*11*.*5±0*.*45*	10.4±0.79	*10*.*4±0*.*73*	10.7±0.55	*9*.*8±0*.*28*

TEP onset latencies (ms) in comparison to the soleus (SOL) H-reflex. MG: medial gastrocnemius; PL: peroneus longus; TA: tibialis anterior; MH: medial hamstring; LH: lateral hamstring; RF: rectus femoris; GRC: gracilis. Based on Student’s *t*-test, *italics* indicate a significant difference on latencies across subject groups.

### Changes in multisegmental motoneuron output after repeated low-frequency transspinal stimulation

**Fig 2:**
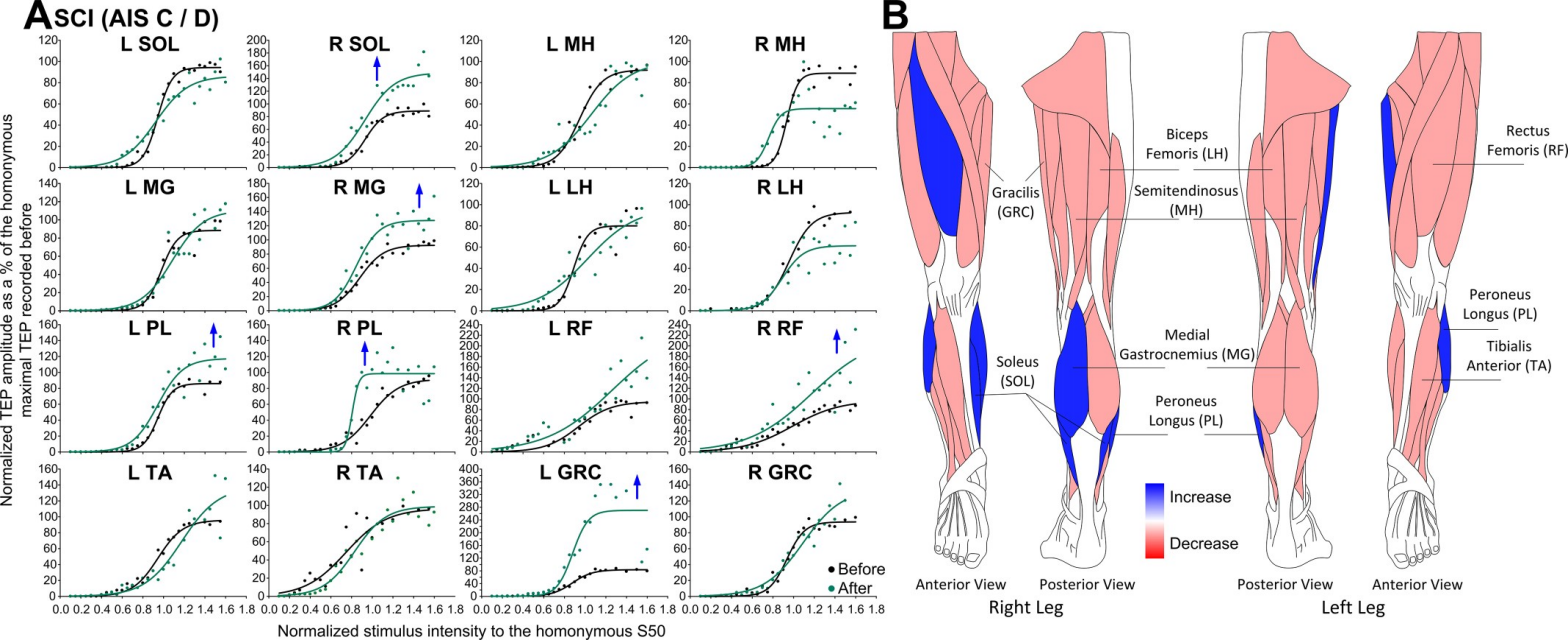
Transspinal evoked potential (TEP) recruitment curves before and after transspinal stimulation in AIS C-D. **(A)** TEPs normalized to the homonymous maximal TEP and plotted against normalized stimulation intensities to the S50-TEPmax obtained before transspinal stimulation training along with the sigmoid function fitted to the data. **(B)** Schematic representation of changes observed in the TEP recruitment input-output curves where *red* represents an increase and *blue* represents a decrease in the corresponding muscle from which the TEP was recorded. SOL: soleus; MG: medial gastrocnemius; TA: tibialis anterior; PL: peroneus longus; MH: medial hamstring; LH: lateral hamstring; RF: rectus femoris; GRC: gracilis. An asterisk indicates a significant difference before and after transspinal stimulation.

The TEP recruitment input-output curves recorded from all muscles along with the associated sigmoid fit for individuals with AIS C-D before and after transspinal stimulation are presented in Fig 2A. TEPs are grouped based on stimulation intensities normalized to the S50-TEPmax obtained at baseline. For clarity purposes, a schematic representation of muscles that changes in the TEP recruitment input-output curves were observed is indicated in Fig 2B. In AIS C-D participants, a significant increase in the TEP input-output curve in right SOL (F_1,174_ = 14.51, *p* < .001), right MG (F_1,189_ = 7.35, *p* = .007), right PL (F_1,131_ = 8.19, *p* = .005), left PL (F_1,144_ = 4.94, *p* = .028), right RF (F_1,138_ = 12.28, *p* < .001), and left GRC (F_1,168_ = 5.19, *p* = .024) muscles was found (Fig 2). These results support for increased responsiveness of ankle and knee extensors motoneurons after repeated transspinal stimulation, functionally related to maintenance of standing posture.

**Fig 3:**
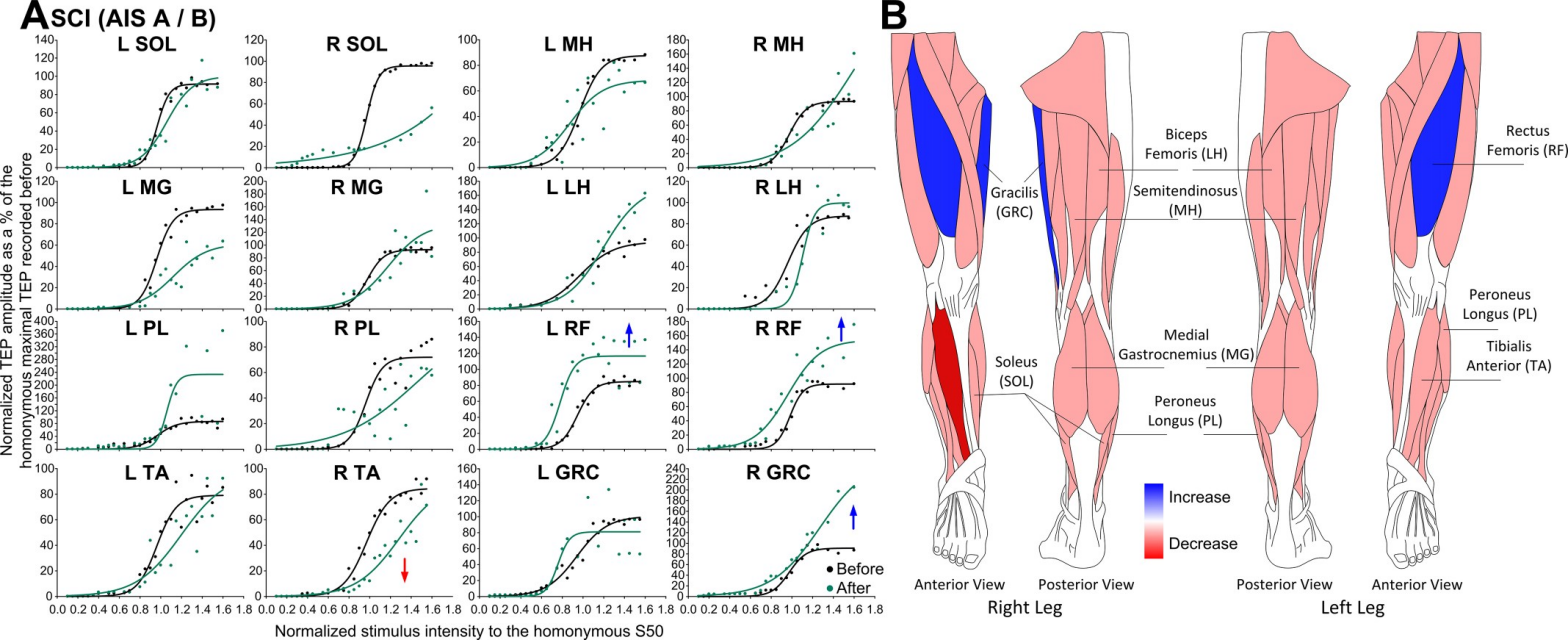
Transspinal evoked potential (TEP) recruitment curves before and after transspinal stimulation in AIS A-B. **(A)** TEPs normalized to the homonymous maximal TEP and plotted against normalized stimulation intensities to the S50-TEPmax obtained before transspinal stimulation training along with the sigmoid function fitted to the data. **(B)** Schematic representation of changes observed in the TEP recruitment input-output curves where *red* represents an increase and *blue* represents a decrease in the corresponding muscle from which the TEP was recorded. SOL: soleus; MG: medial gastrocnemius; TA: tibialis anterior; PL: peroneus longus; MH: medial hamstring; LH: lateral hamstring; RF: rectus femoris; GRC: gracilis. An asterisk indicates a significant difference before and after transspinal stimulation.

The TEP recruitment input-output curves recorded from all muscles along with the associated sigmoid fit for individuals with AIS A-B before and after transspinal stimulation are presented in Fig 3A. TEPs are grouped based on stimulation intensities normalized to the S50-TEPmax obtained at baseline. For clarity purposes, a schematic representation of muscles that changes in the TEP recruitment input-output curves were observed is indicated in Fig 3B. In participants with AIS A-B, a significant increase in the TEP input-output curve in the right RF (F_1,121_ = 11.86, *p* < .001), left RF (F_1,100_ = 31.73, *p* < .001), and right GRC (F_1,86_ = 7.56, *p* = .007) muscles was found after transspinal stimulation (Fig 3A and 3B). Further, the significant decrease in the right TA TEPs (F_1,103_ = 98.15, *p* < .001) coincided with a significant interaction between time and normalized intensities (F_24,103_ = 6.40, *p* < .001). These results suggest that in cases of absent volitional motor control, transspinal stimulation increased motoneuron responsiveness mostly for proximal leg muscles.

**Fig 4:**
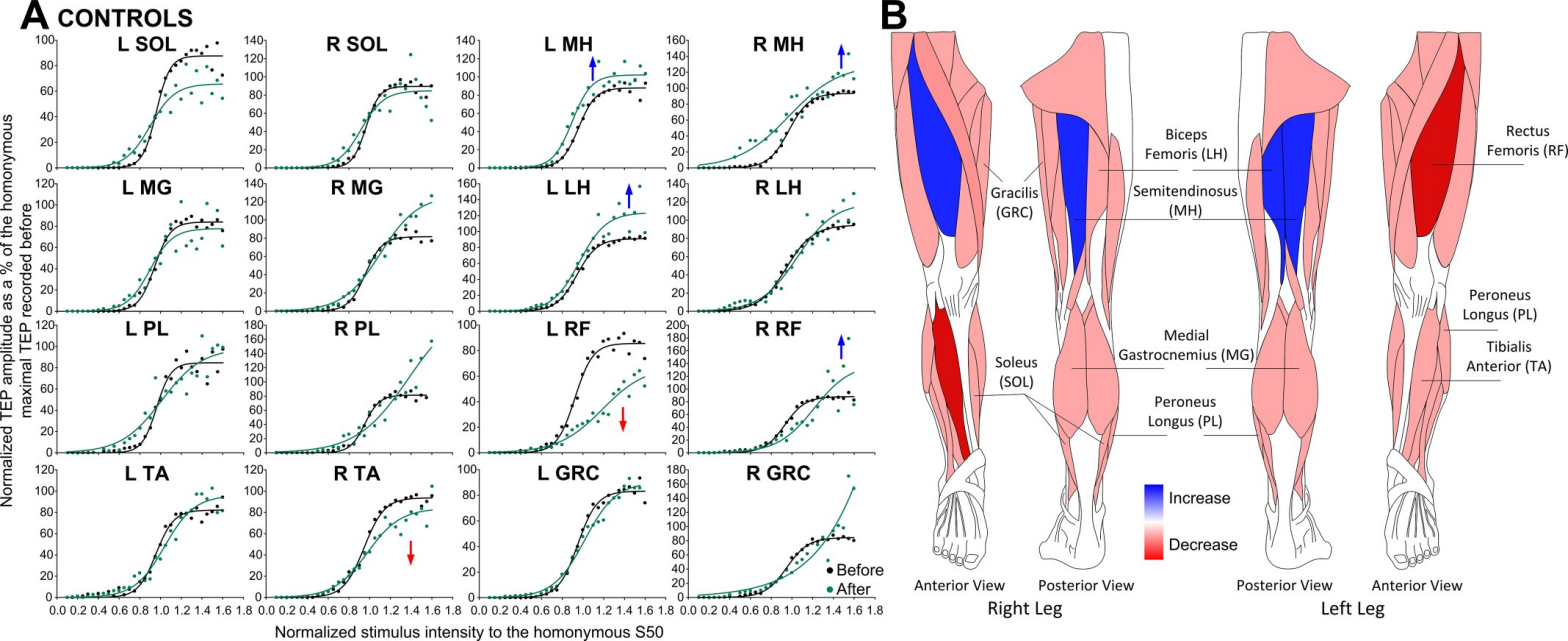
Transspinal evoked potential (TEP) recruitment curves before and after transspinal stimulation in healthy control subjects. **(A)** TEPs normalized to the homonymous maximal TEP and plotted against normalized stimulation intensities to the S50-TEPmax obtained before transspinal stimulation training along with the sigmoid function fitted to the data. **(B)** Schematic representation of changes observed in the TEP recruitment input-output curves where *red* represents an increase and *blue* represents a decrease in the corresponding muscle from which the TEP was recorded. SOL: soleus; MG: medial gastrocnemius; TA: tibialis anterior; PL: peroneus longus; MH: medial hamstring; LH: lateral hamstring; RF: rectus femoris; GRC: gracilis. An asterisk indicates a significant difference before and after transspinal stimulation.

The TEP recruitment input-output curves recorded from all muscles along with the associated sigmoid fit for healthy control subjects are indicated in Fig 4. TEPs are grouped based on time and stimulation intensities normalized to the S50-TEPmax obtained at baseline. Two-way rmANOVA showed that the left MH (F_1,357_ = 14.46, *p* < .001), right MH (F_1,394_ = 8.56, *p* = .004), right RF (F_1,255_ = 23.99, *p* < .001), and left LH (F_1,218_ = 7.295, *p* = .007) TEP recruitment curves and associated maximal values were significantly increased after repeated transspinal stimulation (Fig 4). In contrast, a significant decrease in the right TA (F_1,318_ = 6.33, *p* = .012), and left RF (F_1,145_ = 48.2, *p* < .001) TEPs was found (Fig 4). A significant interaction between time and intensities was found for the right (F_21,255_ = 2.17, *p* = .003) and left (F_20,145_ = 5.93, *p* < .001) RF TEPs. These results support for increased motoneuron output of knee flexor and hip extensor muscles after transspinal stimulation, whilst decreasing motoneuron output in ankle and hip flexors, and knee extensor muscles.

The predicted parameters from the sigmoid function fitted to TEPs plotted against the actual stimulation intensities (recruitment input-output curves) for healthy control subjects only showed a significant decrease in stimulation threshold after repeated transspinal stimulation in the left PL TEP (before: 355.8 ± 73.8 mA, after: 330.5 ± 72.8 mA; *p* = .016), and therefore are not presented in detail. In contrast, for AIS C-D, the *m* function decreased (*p* = .023), the TEP slope increased (*p* = .017), and stimuli for the maximal TEP increased (*p* = .036) for the left MG TEP (Table 3). The *m* function also decreased for the left TA (*p* = .003), suggesting for small selective increased recruitment gains of ankle flexors and extensors in AIS C-D. For AIS A-B, the TEP slope (*p* = .043), S50-TEPmax (*p* = .004), and predicted stimuli at threshold and at maximal intensities decreased (*p* < .05) for the left GRC TEP. The predicted maximum TEP amplitude also decreased for the right SOL (*p* = .022), suggesting for small selective decreased recruitment gains of knee flexors and ankle extensors in AIS A-B.

**Table 3:** Transspinal evoked potential (TEP) recruitment curve sigmoid function parameters before and after transspinal stimulation.

		TEP maximal amplitude		m		Slope		Stimulus threshold		S50		Stimulus max	
		Before	After	Before	After	Before	After	Before	After	Before	After	Before	After
**AIS C / D**													
	L SOL	26.0 ± 4.9	26.8 ± 6.8	0.06 ± 0.02	0.28 ± 0.24	48.7 ± 10.3	60.1 ± 17.2	250.6 ± 36.4	268.2 ± 52.6	299.3 ± 44.4	328.3 ± 68.1	348.1 ± 53.2	388.4 ± 84.2
	R SOL	17.2 ± 2.6	23.7 ± 5.2	0.25 ± 0.14	0.06 ± 0.02	53.1 ± 21.7	63.5 ± 20.5	235.7 ± 29.8	252.2 ± 44.6	288.7 ± 47.2	315.6 ± 57.3	341.8 ± 67.2	379.1 ± 73.6
	L MG	24.9 ± 6.7	33.7 ± 9.6	*0*.*06 ± 0*.*02*	*0*.*03 ± 0*.*02*	*57*.*4 ± 17*.*6*	*109*.*6 ± 26*.*0*	262.2 ± 50.4	311.0 ± 59.9	319.6 ± 63.7	420.7 ± 82.6	*377*.*0 ± 78*.*8*	*530*.*3 ± 107*
	R MG	24.7 ± 2.3	79.9 ± 60.1	0.04 ± 0.01	0.04 ± 0.02	91.1 ± 30.2	92.8 ± 22.0	245.1 ± 34.8	285.2 ± 55.9	336.2 ± 62.5	378.0 ± 72.3	427.2 ± 91.9	470.7 ± 91.0
	L PL	22.1 ± 4.8	26.3 ± 3.3	0.06 ± 0.02	0.04 ± 0.01	52.4 ± 13.9	99.3 ± 34.6	252.4 ± 34.1	274.5 ± 44.4	304.8 ± 46.2	373.9 ± 76.6	357.1 ± 59.1	473.2 ± 110
	R PL	19.6 ± 5.2	18.5 ± 2.7	0.05 ± 0.02	0.05 ± 0.02	124.7 ± 74.2	75.1 ± 20.6	320.0 ± 127	244.9 ± 44.9	444.7 ± 156	320.0 ± 59.5	569.5 ± 209	395.1 ± 77
	L TA	11.4 ± 5.0	8.9 ± 2.2	*0*.*05 ± 0*.*02*	*0*.*03 ± 0*.*01*	61.8 ± 13.1	123.3 ± 39.5	225.2 ± 39.5	259.0 ± 35.4	287.0 ± 42.2	382.3 ± 69.2	348.8 ± 48.4	505.5 ± 107
	R TA	25.0 ± 8.6	10.9 ± 1.7	0.04 ± 0.02	0.05 ± 0.04	83.2 ± 22.1	113.4 ± 29.4	259.1 ± 47.2	251.1 ± 50.6	342.3 ± 67.7	364.6 ± 67.3	425.5 ± 88.9	478.0 ± 90.8
	L MH	66.7 ± 34.3	33.2 ± 8.7	0.05 ± 0.01	0.04 ± 0.01	54.6 ± 11.8	60.8 ± 11.9	228.0 ± 36.6	238.1 ± 23.4	282.6 ± 36.8	299.0 ± 23.2	337.3 ± 40.5	359.8 ± 28.6
	R MH	31.4 ± 6.5	23.6 ± 5.0	0.49 ± 0.43	0.04 ± 0.01	38.9 ± 13.9	76.1 ± 21.3	235.4 ± 25.9	219.2 ± 30.5	274.3 ± 37.1	295.3 ± 39.9	313.2 ± 49.7	371.4 ± 56.2
	L LH	41.7 ± 25.4	19.5 ± 6.3	0.04 ± 0.01	0.04 ± 0.02	62.3 ± 19.6	71.3 ± 20.1	252.4 ± 41.3	232.6 ± 25.7	314.6 ± 51.0	303.9 ± 44.4	376.9 ± 65.4	375.3 ± 64
	R LH	32.7 ± 19.4	15.9 ± 2.9	0.37 ± 0.24	0.04 ± 0.02	79.2 ± 37.1	184.5 ± 94.5	329.5 ± 110	242.5 ± 57.3	408.7 ± 145	427.0 ± 139	487.9 ± 181	611.5 ± 231
	L RF	56.6 ± 39.0	26.9 ± 3.9	0.03 ± 0.01	0.02 ± 0.0	106.6 ± 27.9	132.4 ± 29.8	234.2 ± 36.2	242.5 ± 35.4	340.9 ± 51.9	374.9 ± 41.1	447.5 ± 75.1	507.3 ± 62.5
	R RF	16.4 ± 6.4	20.1 ± 7.6	0.04 ± 0.02	0.01 ± 0.0	109.2 ± 33.7	162.7 ± 28.1	285.1 ± 73.1	234.4 ± 51.7	394.3 ± 100	397.1 ± 54.7	503.5 ± 131	559.8 ± 69.9
	L GRC	21.4 ± 8.1	23.9 ± 4.1	0.03 ± 0.0	0.03 ± 0.01	79.3 ± 12.1	101.7 ± 20.6	229.4 ± 25.2	252.0 ± 27.5	308.7 ± 34.1	353.7 ± 40.6	388.0 ± 44.6	455.3 ± 58.2
	R GRC	15.5 ± 4.1	18.7 ± 4.0	0.12 ± 0.09	0.02 ± 0.01	85.0 ± 36.8	130.9 ± 33.7	251.9 ± 34.5	271.9 ± 34.5	336.9 ± 62.1	402.8 ± 65.4	421.9 ± 96.1	533.7 ± 98.1
**AIS A / B**													
	L SOL	10.8 ± 2.0	23.2 ± 15.0	0.07 ± 0.04	0.08 ± 0.05	53.9 ± 25.0	76.0 ± 27.2	269.4 ± 93.3	388.7 ± 141	323.3 ± 117	464.7 ± 164	377.3 ± 142	540.7 ± 189
	R SOL	*17*.*8 ± 1*.*5*	*7*.*2 ± 3*.*0*	0.12 ± 0.08	0.08 ± 0.03	46.5 ± 20.8	32.3 ± 12.5	311.2 ± 104	249.1 ± 126	357.8 ± 118	281.4 ± 138	404.3 ± 134	313.8 ± 149
	L MG	20.7 ± 6.9	13.4 ± 2.3	0.04 ± 0.03	0.06 ± 0.03	133.4 ± 54.8	71.9 ± 23	512.9 ± 179	384.6 ± 151	646.3 ± 223	456.5 ± 164	779.7 ± 272	528.5 ± 178
	R MG	24.5 ± 7.1	28.9 ± 12.2	1.33 ± 0.93	0.11 ± 0.05	63.0 ± 47.1	75.2 ± 62.4	314.2 ± 103	239.8 ± 99.2	377.2 ± 137	315.1 ± 161	440.2 ± 177	390.3 ± 224
	L PL	17.6 ± 5.4	25.3 ± 12.5	0.07 ± 0.03	0.66 ± 0.64	35.4 ± 14.6	79.0 ± 44.5	321.7 ± 231	266.8 ± 148	357.1 ± 246	345.8 ± 159	392.5 ± 260	424.8 ± 180
	R PL	11.2 ± 1.9	19.9 ± 11.4	0.09 ± 0.06	0.03 ± 0.01	52.2 ± 21.5	105.8 ± 48.3	218.3 ± 91.2	416.0 ± 144	270.5 ± 105	521.8 ± 136	322.7 ± 121	627.7 ± 143
	L TA	6.4 ± 1.3	4.5 ± 1.8	0.06 ± 0.05	0.45 ± 0.43	73.2 ± 63.7	124.5 ± 97.7	394.9 ± 158	235.1 ± 97.4	468.1 ± 179	359.6 ± 195	541.3 ± 218	484.1 ± 292
	R TA	7.2 ± 0.6	7.5 ± 2.4	0.04 ± 0.02	0.02 ± 0.0	80.8 ± 31	134.1 ± 29.4	309.7 ± 108	316.6 ± 123	390.5 ± 121	450.6 ± 150	471.4 ± 139	584.7 ± 177
	L MH	16.2 ± 6.1	12.0 ± 6.1	0.04 ± 0.01	0.03 ± 0.01	69.0 ± 21.3	67.2 ± 11.2	332.2 ± 115	265.9 ± 140	401.1 ± 108	333.1 ± 146	470.1 ± 105	400.3 ± 153
	R MH	18.1 ± 5.0	14.4 ± 4.7	0.04 ± 0.01	0.05 ± 0.02	71.8 ± 19.8	74.8 ± 42.2	353.5 ± 133	337.2 ± 127	425.3 ± 152	412.0 ± 150	497.1 ± 171	486.9 ± 180
	L LH	9.2 ± 4.7	14.6 ± 4.9	0.02 ± 0.01	0.03 ± 0.01	125.3 ± 74.7	78.0 ± 17.5	244.4 ± 139	360.8 ± 129	369.8 ± 214	438.8 ± 145	495.1 ± 288	516.8 ± 161
	R LH	6.9 ± 1.1	13.9 ± 6.1	0.73 ± 0.69	0.04 ± 0.01	30.2 ± 28.79	79.9 ± 40.1	272.5 ± 227	314.4 ± 113	302.7 ± 198	394.3 ± 139	332.9 ± 169	474.2 ± 170
	L RF	18.7 ± 10.3	9.9 ± 2.6	0.03 ± 0.01	0.05 ± 0.02	95.3 ± 37.8	78.2 ± 45.4	318.0 ± 135	157.7 ± 26.7	413.3 ± 151	235.8 ± 72	508.5 ± 173	314.0 ± 117
	R RF	21.1 ± 13.7	11.0 ± 2.1	1.09 ± 0.65	0.18 ± 0.07	6.0 ± 4.4	*13*.*3 ± 5*.*3*	*336*.*6 ± 206*	127.4 ± 27.8	342.6 ± 204	140.6 ± 33.1	348.6 ± 202	153.9 ± 38.4
	L GRC	22.6 ± 6.5	9.1 ± 1.4	0.03 ± 0.01	0.05 ± 0.01	*133*.*6 ± 76*	*37*.*4 ± 4*.*4*	*445*.*1 ± 177*	*129*.*7 ± 20*.*0*	*578*.*7 ± 245*	*167*.*0 ± 15*.*6*	*712*.*3 ± 317*	*204*.*4 ± 11*.*1*
	R GRC	15.5 ± 10.7	15.2 ± 4.1	0.05 ± 0.03	0.04 ± 0.01	62.3 ± 37.8	49.0 ± 9.6	385.9 ± 249	267.6 ± 137	448.1 ± 287	316.6 ± 145	510.4 ± 325	365.6 ± 152

Average predicted parameters from the sigmoid fit to the data for each TEP recruitment curve established separately. *m* is the slope parameter of the function, *S50* is the stimulus required to elicit a TEP equivalent to 50% of the TEPmax. SOL: soleus; MG: medial gastrocnemius; PL: peroneus longus; TA: tibialis anterior; MH: medial hamstring; LH: lateral hamstring; RF: rectus femoris; GRAC: gracilis. Based on Student’s t-test, *boxed italics* indicate a significant effect after transspinal stimulation.

Comparisons between subjects groups showed that before transspinal stimulation, the predicted maximum TEP amplitude was increased in SCI compared to healthy control subjects for the right MG (SCI: 24.6 ± 2.9; controls: 9.5 ± 1.6; *p* < .05), left GRC (SCI: 21.9 ± 5.2; controls: 7.2 ± 1.3; *p* = .014) and right GRC (SCI: 15.5 ± 3.6; controls: 7.0 ± 1.7; *p* = .037). In contrast, the right SOL TEP was markedly reduced (SCI: 17.5 ± 1.6; controls: 27.6 ± 4.2; *p* = .032) for individuals with SCI. After transspinal stimulation, the predicted maximum TEP amplitude was increased in SCI compared to healthy control subjects for the left MG (SCI: 25.6 ± 6.5 ; controls: 10.3 ± 2.7; *p* = .043), right TA (SCI: 9.4 ± 1.4; controls: 4.3 ± 0.9; *p* = .008), left RF (SCI: 21.2 ± 3.9; controls: 4.6 ± 1.4; *p* = .003), left GRC (SCI: 20.2 ± 3.9; controls: 6.1 ± 1.1; *p* = .001) and right GRC (SCI: 17.6 ± 2.9; controls: 5.5 ± 1.2; *p* = .002) muscles. All other predicted parameters were not significantly different from controls.

### Changes in homosynaptic and postactivation TEP depression after repeated low-frequency transspinal stimulation

**Fig 5:**
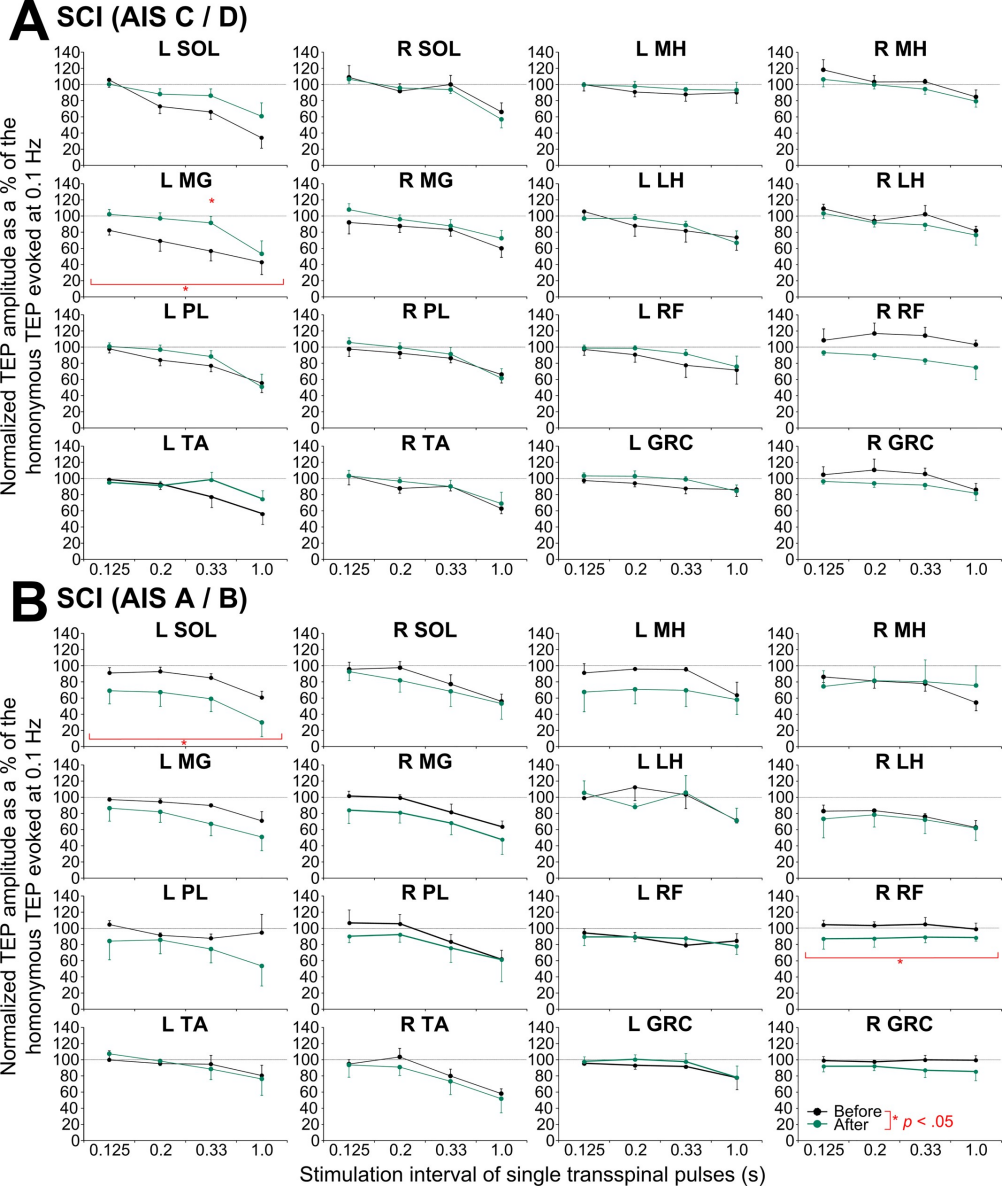
Homosynaptic transspinal evoked potential (TEP) depression before and after transspinal stimulation in spinal cord injury (SCI). Overall percent change of TEPs recorded at 0.125, 0.2, 0.33 and 1.0 Hz from the associated TEP recorded at 0.1 Hz before (black lines) and after (green lines) transspinal stimulation training in individuals with **(A)** AIS C-D and **(B)** AIS A-B. SOL: soleus; MG: medial gastrocnemius; TA: tibialis anterior; PL: peroneus longus; MH: medial hamstring; LH: lateral hamstring; RF: rectus femoris; GRC: gracilis. Error bars indicate SE. An asterisk indicates a significant difference before and after transspinal stimulation.

The average amplitude of normalized TEPs recorded from the left and right leg muscles at different stimulation frequencies grouped for AIS C-D and AIS A-B before and after repeated transspinal stimulation are presented in Fig 5. One-way rmANOVA in AIS C-D for the left SOL TEP recorded at baseline showed a decrease in amplitude as stimulation frequency decreased (F_3,20_ = 8.49, *p* < .001). The same result was also found for TEPs recorded from the left/right TA and left PL muscles, while no significance for the remaining TEPs was found (*p* > .05). This resulted in 4 out of 16 muscles that homosynaptic TEP depression was evident in AIS C-D. In a similar manner, homosynaptic TEP depression was evident in 4 out of 16 muscles in AIS A-B subjects, with TEPs recorded from the left SOL (F_3,10_ = 5.51, *p* = .017), right SOL (F_3,10_ = 4.04, *p* = .04), right TA (F_3,10_ = 6.21, *p* = .012), and right PL (F_3,10_ = 4.62, *p* = .028) muscles to display depression that varied as a function of stimulation frequency at baseline (Fig 5). After transspinal stimulation, homosynaptic TEP depression decreased in the left MG (F_1,36_ = 9.038, *p* = .005) and remained unaltered in the remaining TEPs (Fig 5) in AIS C-D subjects, while homosynaptic TEP depression increased in the left SOL TEP (F_1,21_ = 7.44, *p* = .013) and right RF TEP (F_1,19_ = 6.71, *p* = .018) in AIS A-B subjects.

**Fig 6:**
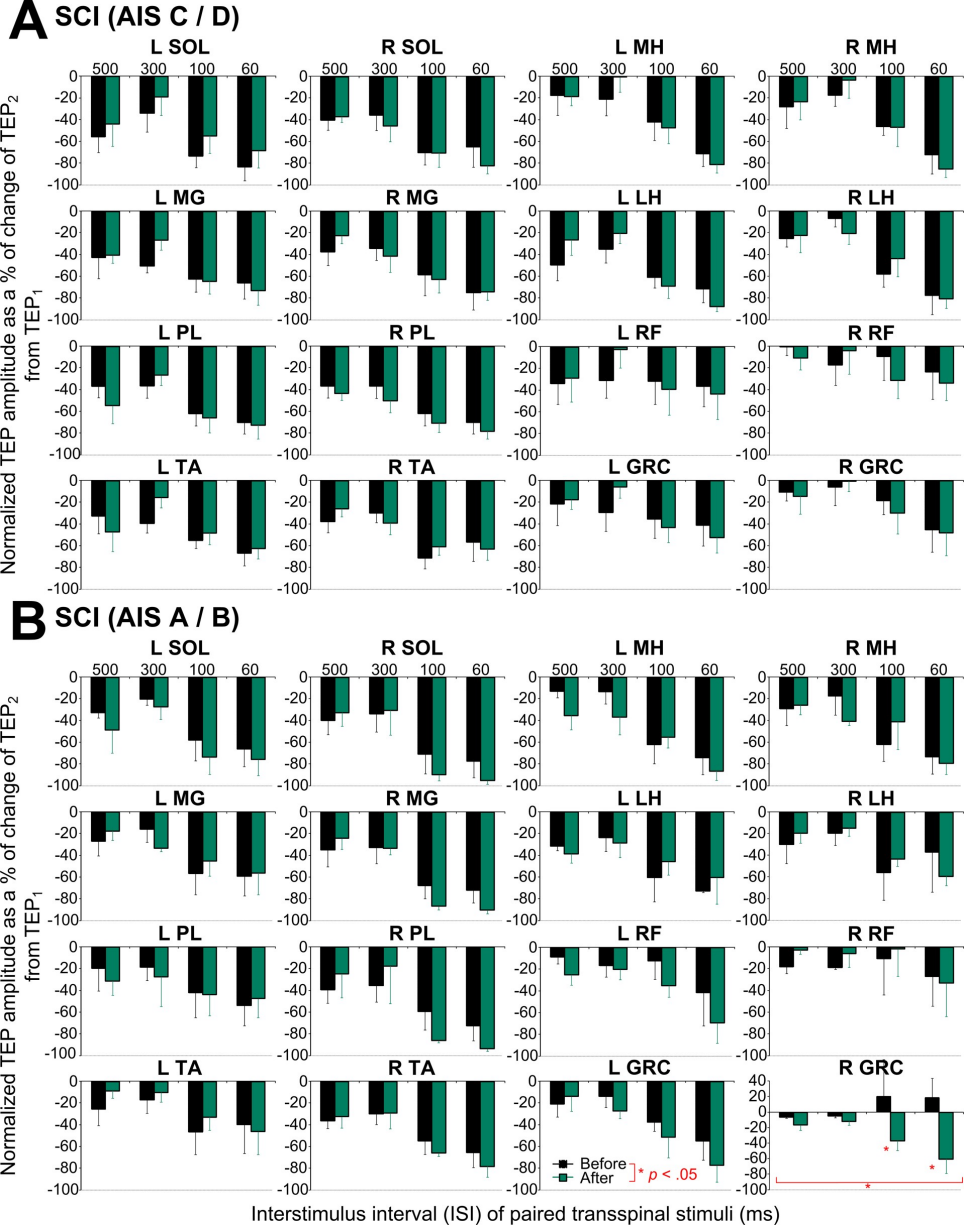
Postactivation transspinal evoked potential (TEP) depression upon paired transspinal stimuli before and after transspinal stimulation in spinal cord injury (SCI). Overall amplitude of the second TEP (TEP_2_) as a percentage of the first mean homonymous TEP amplitude (TEP_1_) evoked at interstimulus intervals of 500, 300, 100 and 60 ms at a constant stimulation frequency of 0.2 Hz for participants with **(A)** AIS C-D and **(B)** AIS A-B. SOL: soleus; MG: medial gastrocnemius; TA: tibialis anterior; PL: peroneus longus; MH: medial hamstring; LH: lateral hamstring; RF: rectus femoris; GRC: gracilis. Error bars indicate SE. An asterisk indicates a significant difference before and after the transspinal stimulation.

The average amplitude of TEPs recorded from left and right leg muscles upon paired transspinal stimuli grouped for AIS C-D and AIS A-B before and after transspinal stimulation are presented in Fig 6. One-way rmANOVA in AIS C-D for the right LH TEP recorded at baseline showed that the amount of depression varied significantly as a function of the ISI tested (H_3_ = 10.38, *p* = .016), while in the remaining TEPs the amount of depression did not vary significantly as a function of the ISI. Similarly, one-way rmANOVA in AIS A-B showed that the amount of depression upon paired transspinal stimuli at baseline varied significantly as a function of ISI only for the left MH TEP (F_3,10_ = 4.67, *p* = .027). After transspinal stimulation, TEP depression upon transspinal paired stimuli remained unaltered in AIS C-D (Fig 6A), and was decreased only in the right GRC TEP (F_1,14_ = 8.31, *p* = .012) in AIS A-B (Fig 6B), suggesting for minimal changes of postactivation depression after transspinal stimulation in individuals with SCI.

**Fig 7:**
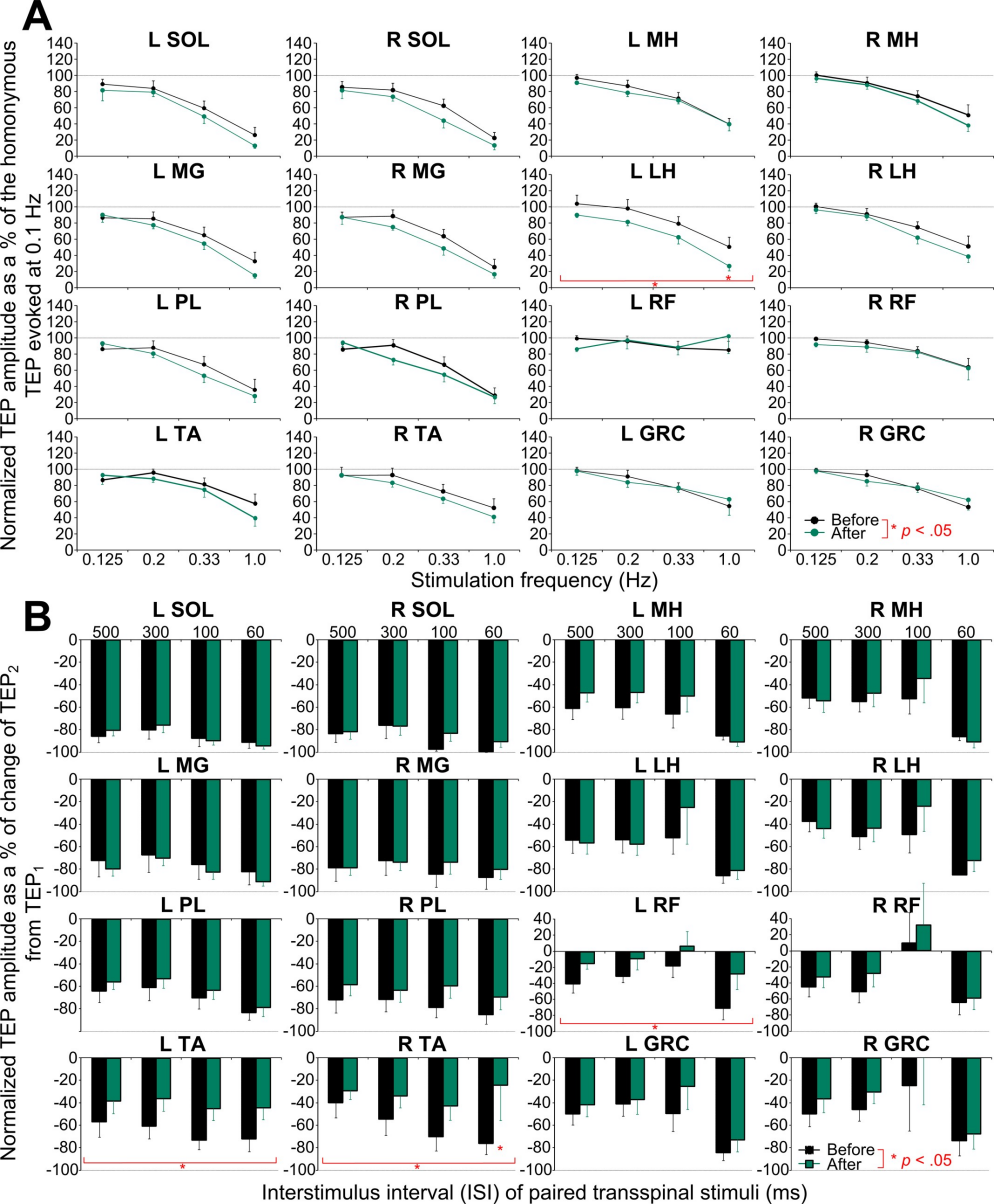
Homosynaptic and postactivation transspinal evoked potential (TEP) depression before and after transspinal stimulation in healthy control subjects. **(A)** Overall percent change of TEPs recorded at 0.125, 0.2, 0.33 and 1.0 Hz from the associated TEP recorded at 0.1 Hz before (black lines) and after (green lines) transspinal stimulation training. **(B)** Overall amplitude of the second TEP (TEP_2_) as a percentage of the first homonymous mean TEP (TEP_1_) evoked at interstimulus intervals of 500, 300, 100 and 60 ms at a constant stimulation frequency of 0.2 Hz. SOL: soleus; MG: medial gastrocnemius; TA: tibialis anterior; PL: peroneus longus; MH: medial hamstring; LH: lateral hamstring; RF: rectus femoris; GRC: gracilis. Error bars indicate SE. An asterisk indicates a significant difference before and after transspinal stimulation.

The group amplitude of normalized TEPs recorded from left and right leg muscles at different stimulation frequencies and upon paired pulses before and after transspinal stimulation in healthy control subjects are indicated in Fig 7. One-way rmANOVA for the left SOL TEP recorded at baseline showed a decrease in amplitude as stimulation frequency decreased (H_3_ = 16.19, *p* = .001). The same result was also found for TEPs recorded from the remaining muscles, except for the left TA (H_3_ = 6.41, *p* = .09) and left RF (H_3_ = 3.99, *p* = .26), suggesting for a generalized susceptibility of TEPs to homosynaptic depression in healthy control subjects. After transspinal stimulation, the amount of homosynaptic depression differed between time (F_1,65_ = 8.70, *p* = .004) and ISI (F_3,65_ = 18.61, *p* < .001) only for the left LH TEP, while no significant changes in the remaining TEPs were found (Fig 7A).

The amount of TEP depression upon paired transspinal stimuli recorded at baseline in healthy control subjects (Fig 7B) did not vary based on the ISI for the left SOL TEP (H_3_ = 5.39, *p* = .15). The same result was found for the left/right MG, left/right TA, right PL, left MH/LH, right RF, and left/right GRC TEPs, and thus resulting in TEPs postactivation depression independent of the ISI used. After repeated transspinal stimulation, the amount of postactivation depression decreased in the left TA (F_1,55_ = 9.997, *p* = .003), right TA (F_1,62_ = 6.03, *p* = .017), and left RF (F_1,47_ = 6.43, *p* = .015) TEPs. These results support that repeated transspinal stimulation affects TEPs homosynaptic and postactivation depression differently in healthy control subjects.

## Discussion

Neuromodulatory noninvasive therapeutic approaches that can strengthen motoneuron depolarization are in great need to promote recovery of motor function after SCI. Here we show for the first time that repeated low-frequency transspinal stimulation over the thoracolumbar enlargement, the location of leg motor circuits, increases the responsiveness of motoneurons over multiple segments in individuals with chronic motor incomplete and complete SCI. While transspinal stimulation induced mixed effects on homosynaptic and postactivation TEP depression, this intervention may be used to functionally improve the neural control of standing and walking.

TEPs had similar latencies and shapes in left and right legs among all subject groups (Fig 1), but more turns within the shape were present in people with SCI, possibly due to less synchronous firing of motor fibers represented by phase cancellation and temporal dispersion in demyelinated nerves [41]. In individuals with SCI, the orderly sigmoidal TEP excitability altered significantly after repeated transspinal stimulation. TEPs recorded mostly at intermediate and maximal intensities were significantly increased largely for the ankle and knee extensors in AIS C-D (Fig 2), while in AIS A-B the increased TEP excitability of knee extensors coincided with decreased excitability of ankle flexors (Fig 3). An increased motoneuron output was also evident for the GRC muscle in the left leg of AIS C-D and right leg in AIS A-B. In the latter case, a significant decrease in the slope of the recruitment curve was evident (Table 3) suggesting altered motoneuronal gain after repeated transspinal stimulation.

While we do not know whether maximal TEPs correspond to the recruitment of the whole motoneuron pool, repeated transspinal stimulation increased the responsiveness of motoneurons that are critical for standing and maintenance of upright posture. This potentiation may have resulted from an increased excitability state enabling alpha motoneurons to produce a more synchronized depolarization. It is possible that the discharge zone of alpha motoneurons residing within the subliminal fringe was expanded after repetitive stimulation of afferents over multiple segments, making motoneurons more easily depolarized to a given input. This is supported by the well-described reversal of subliminal fringe of Renshaw cells upon convergent excitatory inputs [42]. While decreases in threshold intensities based on the sigmoid function were present only in the left GRC TEP in AIS A-B subjects (Table 3), a shift of the TEP recruitment curve to the left was present for the left PL/SOL and right/left MH in AIC C-D (Fig 2A), and right RF and left MH in AIS A-B (Fig 2B). The shift of TEP excitability to the left suggests that repeated low-frequency transspinal stimulation delivered at intensities that motoneuronal depolarization results in bilateral leg extensions followed by subthreshold intensities that may excite interneurons and synapses without direct efferent activity renders the spinal neuronal networks more excitable. A possible mechanism underlying these changes is facilitation of excitatory synapses resulting in long-term potentiation (LTP)-like mechanisms [43]. Because of anatomical and physiological cytoarchitecture differences between the brain and spinal cord, especially after SCI [44], we should be cautious with the general assumption that LTP-mediated plasticity for the spinal cord requires high-frequency stimulation [45,46]. This mechanism is supported by the LTP-induced following low frequency (0.2 Hz) stimulation [47], similar to the frequency we used in this study. Additional evidence supports for LTP-like facilitation mechanisms. For example, transspinal stimulation in midthoracic lateral hemisected rats for 50 min at 0.2 Hz increased the response amplitude of lateral white matter or dorsal corticospinal tracts, and this increase required the activation of *N*-methyl-D-aspartate (NMDA) receptors [29]. Activity of NMDA receptors is known to affect AMPA/kainate receptors which play a crucial role in LTP induction [48–50]. It is possible that, similar to the role described for the silent synapses in LTP [48], repeated transspinal stimulation could have affected the number of motoneurons failing to discharge by raising the firing threshold and thus increasing the size of motoneuronal depolarization [51,52]. Because transspinal stimulation can excite several types of afferents and efferents over multiple segments, it is possible that presynaptic activity may coincide with postsynaptic excitation, which is required for Hebbian LTP [49]. In conclusion, the increased motoneuron output in individuals with chronic motor incomplete and complete SCI after repeated low-frequency transspinal stimulation may be the result of strengthening of spinal synapses via LTP-like mechanisms or via changes in the intrinsic properties of motoneurons. Regardless the underlying mechanism, it is apparent that functionally the increased responsiveness from multiple motoneuron pools can contribute to recovery of motor function, and especially improve standing and walking ability, as reported after epidural stimulation [8,12].

In SCI, a notable contradiction is that although hyperreflexia can produce significant muscle force output it coincides with decreased motoneuronal excitability and volitional motor control [53]. The lack of neuromodulation prevents motoneurons from firing at a frequency sufficient to generate volitional muscle contractions at adequate forces. These results thus strongly support our overall hypothesis that transspinal stimulation alters motoneuron excitability over multiple segments by bringing motoneurons closer to threshold, which is a pre-requisite for functioning descending and local inputs. Additionally, in several cases of complete or incomplete SCI, a single session of transspinal stimulation decreased spasticity, hyperreflexia, and ankle clonus [19,54]. Collectively, repeated transspinal stimulation may be an optimal therapeutic neuromodulation intervention to concomitantly increase motoneuron responsiveness and decrease spasticity. However, spasticity and hyperreflexia over the course of transspinal stimulation requires to be established by neuromechanical studies. Lastly, we should note that repeated transspinal stimulation may be utilized as an adjunct to physical therapy. This proposal is consistent with the reduced spasticity reported after combined interventions including locomotor training and transcranial or transspinal direct current stimulation [55,56].

In individuals with chronic motor complete and incomplete SCI, only 4 out of 16 TEPs portrayed homosynaptic depression (Fig 5) in contrast to the 14 out of 16 TEPs that were susceptible to homosynaptic depression in healthy control subjects (Fig 7A). Based on these results, we suggest that the susceptibility of TEPs to homosynaptic depression depends on presence of volitional motor activity. The soleus H-reflex homosynaptic depression is mediated by consequent activation of the same muscle spindle group Ia afferents, and occurs at the same Ia/motoneuron synapses with absent contribution from spinal inhibitory interneurons or motor axons [57,58]. In the case of TEPs, the decrease of response amplitude as stimulation frequency decreases likely involves spinal inhibitory interneurons spanning multiple segments since TEPs coincide with bilateral muscle contractions resulting in excitation of various afferent types and motor axons [59]. Furthermore, paired transspinal stimuli at ISIs ranging from 60 to 500 ms produced a significant TEP depression. This postactivation depression was similar between subject groups, and was present at baseline in nearly all of TEPs in individuals with chronic motor complete and incomplete SCI (Fig 6) and in healthy control subjects (Fig 7). Repeated low-frequency transspinal stimulation resulted in mixed changes on TEP homosynaptic depression, with both decreases (left MG TEP in AIS C-D; Fig 5A) and increases (left SOL TEP in AIS A-B, Fig 5A and left LH TEP in controls, Fig 7A) being present. Similarly, mixed changes were observed in the TEP postactivation depression, which remained unaltered in AIS C-D (Fig 6A), increased only for one muscle in AIS A-B (right GRC TEP; Fig 6B), and decreased for two muscles in healthy control subjects (left and right TA TEP; Fig 7B). Postactivation depression affects synaptic actions of group I and group II afferents, and low threshold cutaneous afferents synapsing onto intermediate spinal interneurons differently [60]. Based on the mixed changes we observed, different susceptibility of afferents to postactivation depression, and multiple transynaptic actions on spinal neurons as a result of transspinal stimulation, we theorize that in order to postulate changes of homosynaptic and postactivation depression in TEPs recorded bilaterally from knee and ankle muscles, a widespread synaptic plasticity is required, likely related to the number of stimulation sessions.

### Limitations

Transspinal stimulation was delivered with a single, 1-ms square pulse at 0.2 Hz based on previous studies [31,34,36]. We did not use medium or high stimulation frequencies, as we were particularly interested to establish neuroplasticity with low-frequency single pulse stimulation based on its powerful effects on cortical and subcortical neuronal circuits [20,61–63]. Further, we did not perform neurophysiological tests at multiple time points after cessation of stimulation. Thus we are unable to comment on the sustainability of neuroplasticity beyond two days. Lastly, given our sample size and the heterogeneity of SCI [64] we cannot conclude that repeated low-frequency transspinal stimulation is effective for increasing motoneuron responsiveness in all patients. Future studies are warranted to examine different stimulation parameters and time course of neuroplasticity.

## Conclusions

This study provides evidence that repeated transspinal stimulation increases the responsiveness of motoneuron pools over multiple spinal segments, and produces mixed effects in spinal inhibitory mechanisms after motor paralysis or paresis. From a functional perspective, increased motoneuron output may contribute to better motor control and improved tasks such as standing and walking. We propose that noninvasive low-frequency transspinal stimulation can be used to strengthen spinal synapses and depolarization of alpha motoneurons in individuals with chronic motor paralysis or paresis.
